# Molecular detection of *Bartonella* spp. in terrestrial leeches (*Haemadipsa rjukjuana*) feeding on human and animal blood in Gageo-do, Republic of Korea

**DOI:** 10.1186/s13071-016-1613-3

**Published:** 2016-06-07

**Authors:** Jun-Gu Kang, Sohyun Won, Hye-Won Kim, Baek-Jun Kim, Bae-Keun Park, Tae-Seo Park, Hong-Yul Seo, Joon-Seok Chae

**Affiliations:** Laboratory of Veterinary Internal Medicine, BK21 PLUS Program for Creative Veterinary Science Research, Research Institute for Veterinary Science and College of Veterinary Medicine, Seoul National University, Seoul, 08826 South Korea; National Institute of Ecology, 1210 Geumgang-ro, Maseo-myeon, Seocheon-gun, Choongcheongnam-do 33657 South Korea; College of Veterinary Medicine, Chungnam National University, Daejeon, 306-764 South Korea; National Institute of Biological Resources, Incheon, 404-708 South Korea

**Keywords:** *Bartonella grahamii*, *Haemadipsa rjukjuana*, Land leech, Host origin, PCR, Republic of Korea

## Abstract

**Background:**

Leeches can transmit pathogens and are therefore potentially hazardous to human and animal health. However, only a few studies of diseases transmitted by land leeches have been reported. The purpose of the present study was to analyse which pathogens are carried in *Haemadipsa rjukjuana*, the first recorded sanguivorous land leech in the Republic of Korea (ROK).

**Findings:**

A total of 173 *H. rjukjuana* were collected from Mt. Dock-Sil on Gageo-do Island, ROK during July 2011. Conventional PCR was conducted for analysis of the origin of blood meal, as well as for detection of species of *Anaplasma*, *Bartonella*, *Borrelia*, *Ehrlichia*, *Rickettsia*, and *Wolbachia* in the leech specimens*. Bartonella* DNA was detected in eight of the specimens studied based on partial ITS sequence analysis. Seven of the DNA samples were closely related to *Bartonella grahamii* (99.6–100 % similarity), and one sample exhibited a 90.6 % similarity with *Bartonella* sp. from Taiwan. Sequences of the mitochondrial cytochrome *b* gene were generated for a total of 35 of the 173 leech internal organ samples. These included sequences of human (*n* = 10), mouse (*n* = 8), weasel (*n* = 6) and bird (*n* = 11) origin. Of these 35 sequences, 68.5 % were from mammals, including humans, and 31.4 % were from migratory birds that pass through Gageo-do, ROK.

**Conclusions:**

Although the present study does not provide evidence that leeches indeed transmit *Bartonella* species to hosts directly, to our knowledge this is the first report on *Bartonella* DNA being detected from leeches. Therefore, further studies are needed to explore the possibility of zoonotic pathogen transmission by land leeches.

## Background

The possibility that leeches can be vectors of pathogens has been studied in the more recent past, especially with the advent of leech therapy. The medical leech, *Hirudo medicinalis* may bring about severe diseases by transmitting infectious agents that cause syphilis (*Treponema pallidum*), erysipelas (*Streptococcus* sp.), tetanus (*Clostridium tetani*), hog cholera (hog-cholera virus), and hospital wound infection (*Aeromonas hydrophila*) [1–4]. Besides medical leeches, there are reports that *Ozobranchus* (turtle leech) may be a mechanical vector for the fibropapilloma-associated turtle herpesvirus [5] and that *Rickettsia* infection was detected in *Torix tagoi*, *Torix tukubana* and *Hemiclepsis marginata* [6]. These reports suggest that various live bacteria or viruses can remain in the gut of leeches. Although leeches are potentially hazardous to human health, the number of previous studies surveying diseases transmitted by these segmented worms is very limited [7]. Because the blood remaining in the leeches can be detected by polymerase chain reaction (PCR), this method has recently been used to monitor the biodiversity of terrestrial mammals that are blood meal to these worms. Thus PCR is a useful tool for understanding the feeding habits of leeches [8].

In previous studies, *Haemadipsa rjukjuana* (Hirudiniformes: Haemadipsidae) was identified as the first reported sanguivorous land leech in the Republic of Korea (ROK) [9, 10]. Members of the Haemadipsidae are known for their affinity to vertebrate blood [11]. In this study, a survey of prevalent pathogens in *H. rjukjuana* and of its blood meal was conducted by PCR assay. The terrestrial leeches were screened for the presence of *Anaplasma phagocytophilum*, *Anaplasma bovis*, *Ehrlichia chaffeensis*, *Ehrlichia canis*, *Borrelia burgdorferi*, *Bartonella* spp., *Rickettsia* spp. and *Wolbachia* spp. DNA by using a set of species-specific primers. For blood meal screening, the mitochondrial cytochrome *b* gene was used to amplify host blood DNAs from the internal organs of *H. rjukjuana* collected in the field.

## Methods

**Fig. 1:**
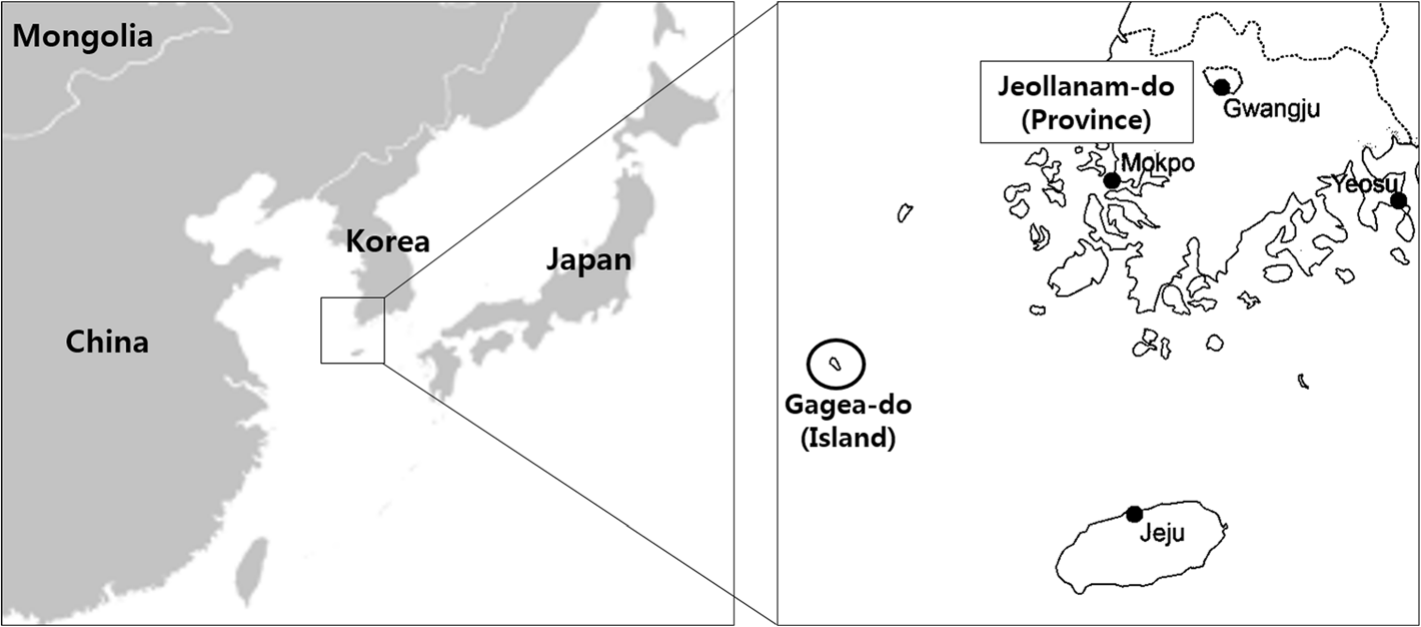
Map of Gageo-do Island in the Republic of Korea. Gageo-do (*black circle*) is at Shinan-gun, Jeollanam-do Province and Korea’s south-westernmost island. Leeches were collected from the mountain Dock-Sil in Gageo-do

**Fig. 2:**
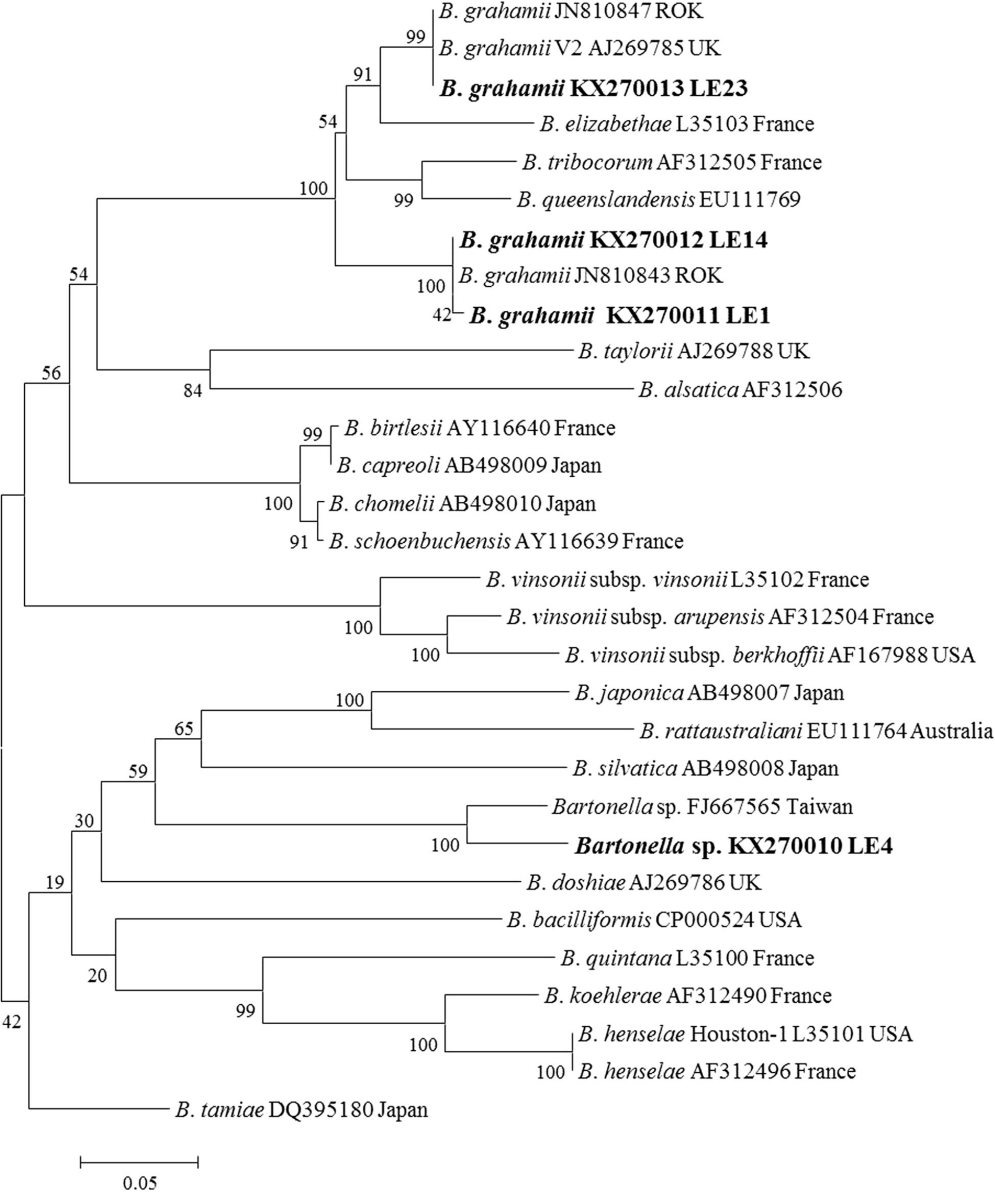
Phylogenetic tree of *Bartonella* spp. detected in blood-feeding terrestrial leeches in Gageo-do based on 16S-23S internal transcribed spacer (ITS) sequences. The neighbor-joining method was used for constructing the phylogenetic tree. The numbers at the nodes are the proportions of 1,000 bootstrap iterations that support the topology shown

A total of 173 terrestrial leeches were collected at Mt. Dock-Sil (altitude 639 m, 34°04'N, 125°07' E) in Gageo-do (Island), Shinan-gun, Jeollanam-do (Province), ROK (Fig. 1) during July 2011. The collection of leeches was conducted by walking along the forest path to attract the leeches and those that attached to the shoes or socks were removed by tweezers as soon as possible. The land leeches were preserved in 70 % ethanol for genomic DNA extraction, which was performed with the DNeasy Blood & Tissue Kit (Qiagen, Hilden, Germany) according to the manufacturer’s instructions and stored at -20 °C. For the detection of zoonotic pathogens, the 16S-23S internal transcribed spacer (ITS) region of *Bartonella* spp., *A. phagocytophilum*, *A. bovis*, *E. chaffensis*, *E. canis*, *B. burgdorferi*, *Rickettsia* spp. and *Wolbachia* spp. were PCR amplified. For analysis of the host animals, the mitochondrial DNA cytochrome *b* gene was amplified by conventional PCR. The primers used for PCR amplification are listed in Table 1. PCR products were separated by electrophoresis in 1.5 % agarose gels and visualised by ethidium bromide staining. The amplicons were analysed by direct sequencing. The GenBank accession numbers of the ITS sequences related to *Bartonella* spp. are shown in Fig. 2. The phylogenetic relationships between haplotypes were reconstructed using the neighbor-joining method under the Maximum Composite Likelihood model. Confidence in the estimated relationship was determined using the bootstrap approach obtained through 1,000 replicates with the same model as mentioned above. Both the bootstrap analysis and the phylogeny reconstruction were conducted using MEGA version 6 [12].

**Table 1:** Nucleotide sequences of polymerase chain reaction primers and conditions used for amplification of *Bartonella* spp. and host genes from land leeches

Target gene	PCR	Name of the PCR primer used	Sequence (5′–3′)	Annealing temperature	Amplicon size (bp)	Reference
*Bartonella* spp. ITS	1^st^	QHVE1	TTCAGATGATGATCCCAAGC	58	735	[21]
		QHVE4	AACATGTCTGAATATATCTTC			
	2^nd^	QHVE12	CCGGAGGGCTTGTAGCTCAG	58	484	
		QHVE14	CACAATTTCAATAGAAC			
*Anaplasma* spp.16S rRNA	1^st^	AE1-F	AAGCTTAACACATGCAAGTCGAA	56	1,406	[21]
		AE1-R	AGTCACTGACCCAACCTTAAATG			
*Anaplasma phagocytophilum* 16S rRNA	2^nd^	EE3	GTCGAACGGATTATTTTTATAGCTTGC	56	926	
		EE4	CCCTTCCGTTAAGAAGGATCTAATCTCC			
*Anaplasma bovis* 16S rRNA	2^nd^	ABkf	TAGCTTGCTATGGGGACAA	59	547	
		ABlr	TCTCCCGCACTCCAGTCTG			
*Ehrlichia* spp. 16S rRNA	1^st^	ECC	AGAACGAACGCTGGCGGCAAGC	56	450	[22]
		ECB	CGTATTACCGCGGCGCTGGCA			
*Ehrlichia chaffeensis* 16S rRNA	2^nd^	HE3	TATAGGTACCGTACTTATCTTCCCTAT	56	390	
		HE1	CAATTGCTTATAACCCTTTTGGTTATAAAT			
*Ehrlichia canis* 16S rRNA	2^nd^	HE3	TATAGGTACCGTACTTATCTTCCCTAT	56	365	
		ECAN5	CAATTATTTATAGCCTCTGGCTATAGGC			
*Borrelia burgdoferi* 16S rRNA	1^st^	B1	CAGTGCGTCTTAAGCATGC	59	1,427	[21]
		B8	CCTTAAATACCTTCTCCC			
	2^nd^	B3	GCAGCTAAGAATCTTCCGCAATGG	60	714	
		B6	CAACCATGCAGCACTGTATAT			
*Rickettsia* sp. *glt*A	1^st^	RpCS. 877p	GGGGACCTGCTCACGGCGG	54	382	[23]
		RpCS. 1258n	ATTGCAAAAAGTACAGTGAA			
	2^nd^	RpCS. 896p	GGCTAATGAAGCAGTGATAA	58	338	
		RpCS. 1233n	GCGACGGTATACCCATAG			
*Wolbachia* 16S rRNA		16S-F	TTGTAGCCTGCTATGGTATAACT	56	937	[24]
		16S-R	GAATAGGAGTTTTCATGT			
mt DNA cytochrome *b* gene		L14841	AAAAAGCTTCCATCCAACATCTCAGCATGATG	50	450	[25]
		H15149	AAACTGCAGCCCCTCAGAATGATATTTGTCCTCA

## Results

The PCR amplification of the partial ITS sequences resulted in detection of *Bartonella* spp. from 8 of the 173 leech specimens. Of these, seven samples were closely related to *Bartonella grahamii* with 99.6–100 % sequence similarity and one sample exhibited a 90.6 % similarity with *Bartonella* sp. KM2563 (FJ667565) from a wild rodent in Taiwan. Among the seven *B. grahamii* sequences, two were closest to *B. grahamii* KWDBG 41 (JN810847, ROK) from *Apodemus agrarius* (99.6–100 % similarity, respectively), and the other five samples were identical with *B. grahamii* V2 (AJ269785). As a result, the sequences of *B. grahamii* obtained in this study were divided into two sub-clades. One sub-clade included the V2 isolate from the United Kingdom (AJ269785) and one Korean genotype (JN810847) and the other sub-clade included another Korean genotype (JN810843) (Fig. 2). These results suggest that *B. grahamii* may have two different strains existing in the ROK.

With regard to the mitochondrial DNA cytochrome *b* gene analysis to identify host animals, a total of 35 sequences were obtained from the 173 leech samples. The rest of the samples were not amplified or proved to be of other species. Of the 35 amplicons analysed, human (*n* = 10), mouse (*n* = 8), weasel (*n* = 6), pale thrush (*n* = 3), grey-backed thrush (*n* = 3), rufous-tailed robin (*n* = 1), Siberian rubythroat (*n* = 1), oriental magpie robin (*n* = 1), black-faced bunting (*n* = 1) and yellow-throated bunting (*n* = 1) DNA sequences were detected (Table 2). About 68.5 % of the sequences were mammalian, including human, in origin, and about 31.4 % were from migratory birds that pass through Gageo-do Island. Only a single DNA sequence of weasel origin was detected from leeches positive for *Bartonella* spp.

**Table 2:** Detection of host genes using mtDNA cytochrome *b* gene from land leeches

Detected hosts	Scientific names	Category	No. of identified samples
Human	*Homo sapiens*	Mammal	10
Mouse	*Mus musculus*	Mammal	8
Weasel	*Mustela sibirica*	Mammal	6
Pale ouzel	*Turdus pallidus*	Bird	3
Grey-backed thrush	*Turdus hortulorum*	Bird	3
Rufous-tailed robin	*Luscinia sibilans*	Bird	1
Siberian rubythroat	*Luscinia calliope*	Bird	1
Oriental magpie robin	*Copsychus saularis*	Bird	1
Black-faced bunting	*Emberiza spodocephala*	Bird	1
Yellow-throated bunting	*Emberiza elegans*	Bird	1
Total			35

## Discussion

Most vector-borne diseases, including dengue, Lyme disease, malaria, endemic typhus, and bartonellosis are transmitted by sucking arthropods such as fleas, mosquitoes and ticks [13]. Leech is also a haematophagous ectoparasite, sucking the blood of humans and animals such as fishes, frogs, turtles and birds. The process of digestion of the ingested blood is very slow so that the blood can remain in the leech gut for up to 27 weeks [7, 14]. In fact, researchers have recently suggested that leeches might be promising candidates as vectors [8]. Reports have been published on infection of leeches by *Streptococcus* spp., *Clostridium tetani*, classical swine fever virus, *Aeromonas hydrophila*, bovine parvovirus, feline calicivirus, equine arteritis virus, equine herpesvirus type 1, and *Rickettsia* spp. [1–4, 7]. In the present study, both *Bartonella* sp. and *B. grahamii* were detected from DNA extracted from the leeches, with a total infection rate of 4.6 % (*n* = 8/173). The 16S-23S ITS region of *Bartonella* spp. was chosen for the PCR amplification, being a useful genetic marker to identify this species because of its hyper-variable sequences and much lower sequence similarity than other target genes [15].

*Bartonella* spp. are small, gram-negative bacteria that infect red blood cells and invade endothelial cells, and are infective to hosts or reservoirs. Several *Bartonella* spp. are identified as zoonotic agents causing cat scratch disease, bacillary angiomatosis, neuroretinitis, etc. [16]. Because most of the species of *Bartonella* have been detected or cultured from numerous arthropods, various arthropods have been explored as potential vectors for this bacterial species [17], with ticks being a representative example considering some recent reports providing evidence that *Ixodes ricinus* may serve as a competent vector for *Bartonella* spp. suggesting a potential new vector [18, 19]. However, its biological role has to be further investigated.

*Bartonella* spp. also have a natural cycle that comprises a vector and a host as other pathogens. Although our data was only represented the molecular evidence of *Bartonella* in leeches, we think that leech may serve as a host for *Bartonella* species. In addition to the record of *Bartonella* spp., a study of the blood meal is very important toward understanding the host preferences and vector capacity of leeches. Furthermore, blood meal is commonly used to investigate the distribution of hosts and to control disease spreading [8, 20]. In the present study, human, mouse, weasel and bird DNA was detected from the terrestrial leeches inhabiting Mt. Dock-Sil (Table 2). Additionally, one DNA sample of weasel origin was identified from a leech positive for *B. grahamii*. This finding indicates that these leeches are likely to carry the blood of animals and thereby disperse several pathogens.

Because Gageo-do is an isolated island far from the mainland (Fig. 1), the mammalian fauna on the island is very sparse. Although only a few mammal species were detected as hosts in the blood meal, many bird species were evident. Gageo-do is known as a major stopover location for migratory birds that pass through the Yellow Sea; hence, the bird species detected in this study are all migratory birds that fly in from the north. Recently, the faunal distribution has been greatly influenced by rapid climate change, increasing the chance of inflow of new vectors and diseases. Blood meal screening could track the change of migratory birds that stop over at Gageo-do as well as their current distribution.

Our study provided new data on the potential role leeches may play in *Bartonella* spp. transmission. Although it does not prove that leeches indeed transmit the bacteria to the hosts, to our knowledge, this is the first report of *Bartonella* DNA being detected from leeches. The vector competence is completed when the zoonotic agent is transferred from the vector to the host reliably, and vertical or horizontal transmission should be possible [6, 17]. Therefore, further studies are needed to prove the transmission of zoonotic pathogens by land leeches.

## Abbreviations

DNA, deoxyribonucleic acid; ITS, internal transcribed spacer; PCR, polymerase chain reaction; ROK, Republic of Korea
